# Acupuncture for patients with mild cognitive impairment: a randomized, patient–assessor-blinded, sham-controlled pilot study

**DOI:** 10.1186/s12906-025-05023-5

**Published:** 2025-07-19

**Authors:** Yujin Choi, Pyung-Wha Kim, In-Chul Jung, Ae-Ran Kim, Hyo-Ju Park, Ojin Kwon, Jun-Hwan Lee, Joo-Hee Kim

**Affiliations:** 1 KM Science Research Division, Korea Institute of Oriental Medicine, Daejeon, Republic of Korea; 2 R&D Strategy Division, Korea Institute of Oriental Medicine, Daejeon, Republic of Korea; 3 Department of Neuropsychiatry, College of Korean Medicine, Daejeon University, Daejeon, Republic of Korea; 4 Department of Acupuncture and Moxibustion Medicine, College of Korean Medicine, Sangji University, 83 Sangjidae-gil, Wonju, 26339 Republic of Korea; 5 Research Institute of Korean Medicine, Sangji University, Wonju, Republic of Korea

**Keywords:** Acupuncture therapy, Alzheimer’s disease, Cognitive dysfunction, Dementia, Randomized controlled trial

## Abstract

**Background:**

Mild cognitive impairment (MCI) is the transitional stage between normal aging and early dementia. Although acupuncture is a promising non-pharmacological treatment, higher-quality evidence is needed to confirm its effectiveness.

**Methods:**

A randomized, patient- and assessor-blinded, sham-controlled, pilot clinical trial was conducted to evaluate the feasibility of acupuncture for treating MCI. In total, 30 participants were randomized into acupuncture and sham acupuncture groups. The participants underwent 24 treatment sessions over 12 weeks. The primary outcome was a change in the Alzheimer’s Disease Assessment Scale-Cognitive Subscale (ADAS-cog) score over 12 weeks, whereas the secondary outcomes included the Seoul Neuropsychological Screening Battery (SNSB-II) score. Adverse events and the success of blinding were also assessed.

**Results:**

Of the 30 participants, 28 completed the study. Participants in the acupuncture and sham acupuncture groups exhibited a decrease in ADAS-cog scores from 10.27 ± 4.03 and 11.47 ± 3.85 at baseline to 5.78 ± 3.04 and 6.27 ± 2.83 at week 12, respectively. Both groups exhibited clinically meaningful improvements, with no significant difference between groups (*P* = 0.6590). The SNSB-II memory domain exhibited a moderate effect size favoring acupuncture (Cohen’s d = 0.57, *P* = 0.1317). No intervention-related adverse events were reported, and participant blinding was adequate throughout the trial.

**Conclusions:**

The 12-week acupuncture treatment is feasible for patients with MCI and may improve memory. Although the primary outcomes did not reach statistical significance, the secondary outcomes suggested potential benefits. Larger confirmatory trials are warranted to investigate the effectiveness of acupuncture in patients with MCI.

**Trial registration:**

Clinical Research Information Service (cris.nih.go.kr) KCT0001938 (Registered on June 3, 2016).

**Supplementary Information:**

The online version contains supplementary material available at 10.1186/s12906-025-05023-5.

## Background

Mild cognitive impairment (MCI), a transitional state between normal cognition associated with aging and early dementia, is characterized by memory loss to the extent higher than that is considered normal with aging but with relatively intact function in other cognitive domains [1, 2]. Approximately 10–15% of patients with amnestic MCI progress to annually, with more than half experiencing AD within 5 years [3, 4]. Previous epidemiological studies have reported 3–19% MCI prevalence in older adults aged > 65 years [4]. Notably, the prevalence of dementia increases exponentially with age, doubling every 5 years after 65 years of age [5]. Detecting and managing MCI in its early stages is crucial, as MCI is a high-risk factor for developing dementia, and most individuals who visit clinics for memory disorders likely already have dementia [3].

Acupuncture is a promising non-pharmacological treatment for cognitive impairment [6]. Acupuncture induces changes in brain areas such as the cingulate cortex, prefrontal cortex, and hippocampus in patients with MCI [7]. An overview of systematic reviews published in 2021 suggested that acupuncture may be effective for MCI and dementia [8]. A systematic review published in 2023 reported that, compared with conventional medicine or sham acupuncture, acupuncture better improved overall cognitive function in patients with MCI [9]. Recent meta-analyses have further reported the efficacy and safety of acupuncture for cognitive impairment [10] and its modulatory effects on brain regions associated with memory and cognition [11]. However, this systematic review also reported a high risk of bias in many included studies and highlighted the requirement for high-quality evidence. Additionally, a study evaluating the reporting quality of clinical trials on acupuncture for MCI revealed low to moderate quality of published reports, indicating room for improvement [12].

Although acupuncture exhibits potential as a treatment for MCI, the existing evidence remains insufficient because of the high risk of bias and low reporting quality of previous studies. Before conducting large-scale confirmatory trials, pilot studies are essential to establish feasibility including recruitment processes, intervention implementation, and data collection procedures. Therefore, this pilot study aimed to investigate the feasibility of a rigorously designed acupuncture trial for MCI, with secondary exploration of preliminary effectiveness and safety to provide data for future confirmatory research.

## Methods

### Study protocol

The study protocol was approved by the Institutional Review Board of the Daejeon Korean Medicine Hospital of Daejeon University (approval number: djomc-131-1). All participants provided written informed consent before screening. The trial was registered with the Clinical Research Information Service under registration number KCT0001938 on June 3, 2016 (https://cris.nih.go.kr/cris/search/detailSearch.do?seq=5900). The study protocol was registered after the first participant was enrolled, and no changes were made to the research plan during the study period. The results of this clinical trial are reported in accordance with the CONSORT guidelines for randomized controlled trials [13], STRICTA checklist for acupuncture trials [14], and ACURATE checklist for sham acupuncture trials [15]. Additional files - Supplementary materials 1, 2, and 3.

### Study design

This was a randomized, patient- and assessor-blinded, sham-controlled, pilot clinical trial with two parallel groups—an acupuncture group and a sham acupuncture group—with an allocation ratio of 1:1. The trial was conducted at two Korean medicine university hospitals in Daejeon, Republic of Korea.

### Participants

#### Inclusion criteria

Patients with MCI aged ≥ 50 years were recruited between January 2016 and September 2016. Participants were screened at the study sites to ensure that they met the following inclusion criteria: patients who met the Peterson diagnostic criteria for MCI [16] with memory problems for at least 3 months, a Clinical Dementia Rating score of 0.5 [17], a Global Deterioration Scale (GDS) score of 2 or 3 [18], a Hachinski Ischemic Score of < 4 [19], at least 6 years of education, and provided written informed consent.

#### Exclusion criteria

The exclusion criteria were as follows: dementia diagnosed based on the Diagnostic and Statistical Manual of Mental Disorders-IV; history of cognitive impairment due to other causes such as head trauma or brain injury; history of cerebral hemorrhage or infarction; Parkinson’s disease, normal pressure hydrocephalus, brain tumor, or cerebrovascular disease; psychiatric illnesses, including schizophrenia, delusional disorder, or bipolar disorder; severe medical diseases, such as diabetic complications, cardiovascular, hepatic or renal disorders, or malignancy; anemia, hypothyroidism, or vitamin deficiencies; history of drug or alcohol dependence in the past 6 months; currently on medication for cognitive function; received any treatment for MCI within the past 2 weeks; illiterate; participation in other clinical trials within the past 4 weeks; known hypersensitivity to acupuncture treatment or inability to cooperate with the acupuncture procedure; being pregnant, expecting to be pregnant, or lactating; difficulty adapting to the treatment visits or answering the questionnaire; and unwilling to comply with the study protocol.

### Randomization and blinding

Eligible participants were randomized into two groups using block randomization to ensure balanced sample sizes across the groups over time. Random sequences were generated by an independent statistician using SAS^®^ Version 9.4 (SAS Institute Inc., Cary, NC, USA). Random numbers were assigned to the participants sequentially, and allocation according to the random number was concealed using sealed opaque envelopes. After a random number was assigned, the corresponding sealed envelope was opened, and participants were allocated to their respective groups.

As this trial was both participant- and assessor-blinded, outcome assessments were performed by investigators who were not involved in the randomization process or the administration of acupuncture treatments. The assessors asked only simple and necessary questions to prevent patients from deducing their treatment group. The assessments were conducted in separate rooms from treatment areas, and assessors had no access to treatment allocation information. For patient blinding, participants were informed that they would receive one of two types of acupuncture treatments: “classical acupuncture” or “non-classical acupuncture.” The success of participant blinding was evaluated using the blinding index and credibility assessment as reported in Sect. 2.9.

### Interventions

#### Acupuncture

Participants in the acupuncture group received standardized acupuncture treatment by a Korean medicine doctor with at least 2 years of clinical experience. Based on previous research [20–22], 14 acupuncture points—GV20, EX-HN1, CV12, and the bilateral points of ST36, HT7, KI3, and SP6—were selected with consensus from five Korean medicine doctor specialists. All acupoint location were determined according to the WHO Standard acupuncture point locations in the western pacific region [23]. Sterile, disposable acupuncture needles (stainless steel, size 0.25 × 40 mm; Dongbang Medical Co., Ltd., Republic of Korea) were used. Manual stimulation was applied to elicit a “de-qi” sensation, defined as a feeling of heaviness around the acupoint. Each participant received 24 treatment sessions over 12 weeks (twice a week), with the needles retained for 30 min per session. Absence for up to 25% of the total treatment sessions was permitted.

#### Sham acupuncture

**Fig. 1:**
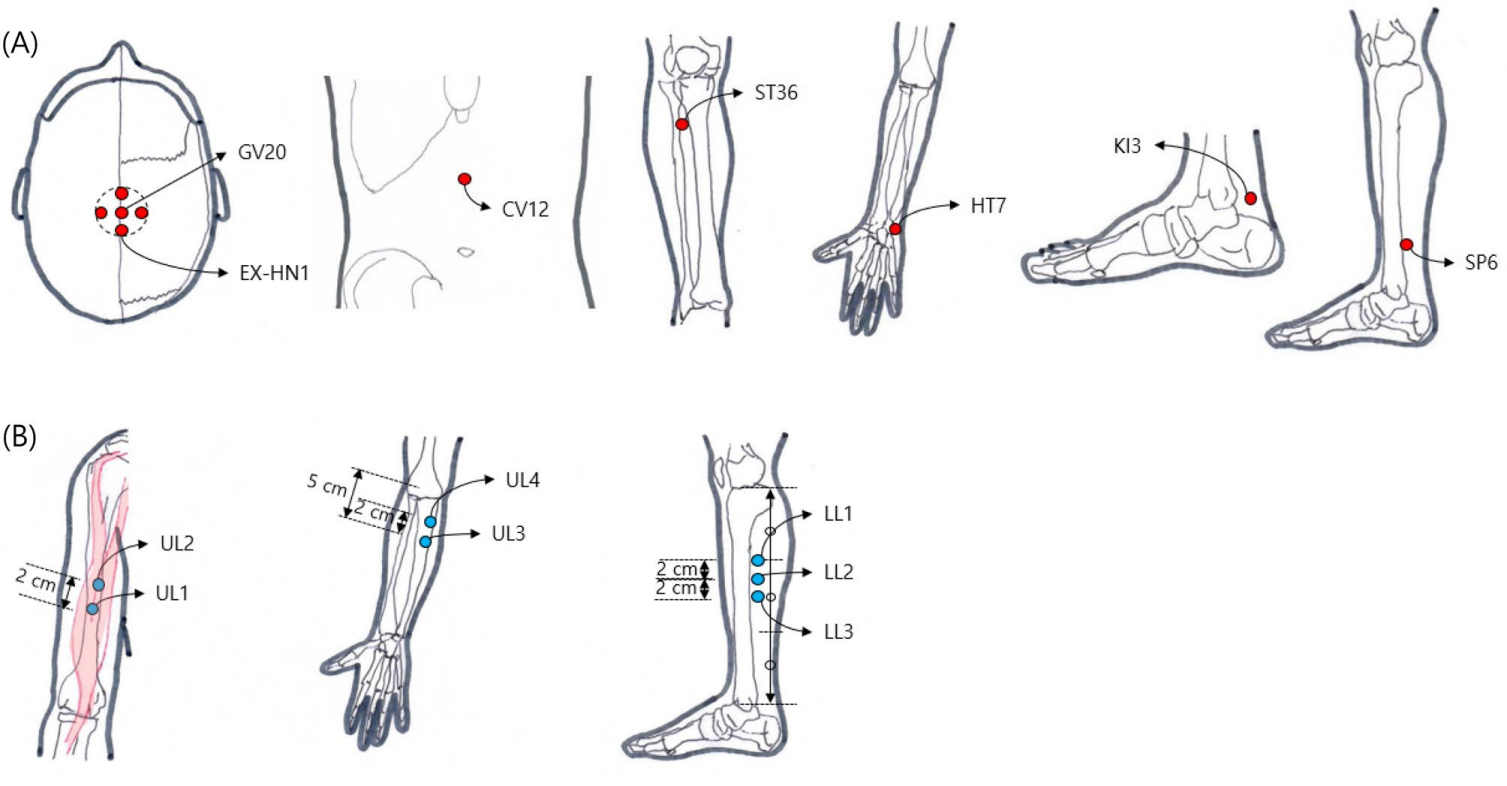
Locations of points used in the acupuncture (**A**) and sham acupuncture group (**B**)

Participants in the sham acupuncture group received sham acupuncture treatment at 14 sham acupoints using a non-penetrating Park sham device [24]. Sham acupoints were selected based on the following criteria to minimize confounding effects: (1) locations that do not correspond to recognized traditional acupoints; (2) areas without established therapeutic effects for cognitive function in the literature; and (3) considering that dermatome overlap between verum and sham acupuncture can influence clinical outcomes [25], dermatome overlap was minimized where anatomically feasible. The specific locations were as follows: the midpoint of the biceps brachii muscle belly (upper extremity 1, UE1), 2 cm above UE1 (UE2), 5 cm below the elbow crease, 1 cm lateral (UE3), 2 cm above UE3 (UE4), the upper third of the medial part of the tibia (lower extremity 1, LE1), 2 cm below LE1 (LE2), and 2 cm below LE2 (LE3), all bilaterally. The locations of points used in the acupuncture and sham acupuncture group are presented in Fig. 1. The other treatment procedures were the same as those used in the acupuncture group. Participants in both groups were prohibited from receiving other treatments for MCI during the trial period.

### Outcome measures

#### Primary outcome

The primary outcome was the Korean version of the Alzheimer’s Disease Assessment Scale-Cognitive Subscale (ADAS-cog) [26]. The ADAS-cog consists of 11 subtests, including memory items (word recall, orientation, word recognition, and remembering word recognition test instructions), five language items (naming, word-finding ability, commands, spoken language ability, and comprehension of spoken language), and two performance items (constructional praxis and ideational praxis) [27]. The total score ranges from 0 to 70, with a higher score indicating greater cognitive impairment, as it represents the sum of errors. The ADAS-cog was assessed at baseline and at 4, 8, and 12 weeks after treatment. The primary outcome was the total ADAS-cog score at the end of the 12-week treatment period.

#### Secondary outcomes

The secondary outcomes included the ADAS-cog scores at 4 weeks and 8 weeks, the Korean version of the Montreal Cognitive Assessment (MoCA) [28], the 2nd edition of the Seoul Neuropsychological Screening Battery (SNSB-II) [29], and the Patient Global Impression of Change (PGIC). The MoCA is a brief cognitive screening tool with high sensitivity and specificity for detecting MCI, with a total score from 0 to 30, wherein a higher score indicates less impaired cognitive function. It includes items assessing visuospatial and executive functions, naming, attention, language, abstraction, memory, and orientation [30]. The MoCA was assessed at baseline and at 4, 8, and 12 weeks after treatment.

The SNSB-II is a standardized neuropsychological test battery widely used in Korea to assess cognitive functioning [29]. It includes various cognitive tasks for evaluating five cognitive domains: attention, memory, language, visuospatial function, and frontal/executive function. For example, it uses the digit span test for attention, the Boston Naming Test and calculation tasks for language and related functions, the clock drawing test and Rey Complex Figure Test (RCFT) [31] for visuospatial functions, the Seoul Verbal Learning Test Elderly’s version (SVLT-E) [32] for memory, and the Color Word Stroop Test and Controlled Oral Word Association Test for frontal/executive function. The SNSB was assessed at baseline and at 12 weeks after treatment.

The PGIC is a self-assessment tool that allows patients to assess perceived improvement after treatment. Patients can select one of seven options to evaluate how their MCI symptoms have changed: “Very much improved,” “Much improved,” “Minimally improved,” “No change,” “Minimally worse,” “Much worse,” or “Very much worse.” The PGIC was assessed at baseline and at 12 weeks after treatment.

All assessment data were collected using an electronic case report form. Data were initially recorded by investigators, and a clinical research associate performed full source data verification for all inputted data.

### Sample size calculation

This pilot clinical trial aimed to evaluate the feasibility of acupuncture treatment for MCI. Notably, a minimum of 12 participants per group are recommended for pilot studies [33]. Considering the number of participants that can be recruited, the minimum range for evaluation, and a dropout rate of 20%, the total number of participants was calculated to be 30.

### Adverse events

Participants were instructed to report any adverse event as it occurred. The investigator monitored the occurrence of adverse events through laboratory test results and interviews. When an adverse event occurred, the date of onset and resolution, the severity and outcome of the event, any action taken related to the intervention, the causality with the intervention, any suspected medication or treatment other than the intervention, and details of any treatment for the adverse event were recorded.

### Blinding test

To assess the success of blinding, participants were asked to guess which treatment they received after the first and last interventions [34], with the following options: “classical acupuncture,” “non-classical acupuncture,” or “don’t know which treatment I received.” Additionally, the credibility of the treatment was evaluated at the end of the 12-week treatment period [35]. Participants rated their responses to the following four questions: “How confident do you feel that this treatment can alleviate your complaint?” “How confident would you be in recommending this treatment to a friend who suffered from similar complaints?” “How logical does this treatment seem to you?” and “How successful do you think this treatment would be in alleviating other complaints?” using a 7-point Likert scale (0 = very low to 6 = very high).

### Statistical analysis

The primary and secondary outcomes were analyzed using an intent-to-treat dataset. However, participants with predefined major protocol violations, such as violating the inclusion/exclusion criteria, not receiving the intervention even once, or not having undergone at least one primary outcome assessment after randomization, were excluded from the analysis. For the primary outcome analysis, the null hypothesis was that there would be no difference in the ADAS-cog scores between the acupuncture and sham acupuncture groups 12 weeks after baseline. This was tested using an analysis of covariance with the 12-week ADAS-cog score as the dependent variable, the group as the fixed factor, and the baseline ADAS-cog score as the covariate. Missing values were addressed using multiple imputations. Continuous outcomes, such as MoCA and SNSB scores, were analyzed using the same method. The results of the PGIC, blinding test, and credibility assessment were analyzed using the chi-square or Fisher’s exact test. Additionally, repeated measures ANOVA was performed to evaluate changes across all time points (baseline, 4 weeks, 8 weeks, and 12 weeks) for ADAS-cog and MoCA scores.

Safety analysis was conducted using a dataset comprising participants who received the intervention at least once. The frequencies of adverse events, adverse events related to the intervention, and serious adverse events were summarized for both groups.

The baseline characteristics of the participants in the two groups are presented as the mean ± standard deviation or frequency (%). Comparisons between groups were performed using the t-test or Wilcoxon rank-sum test for continuous variables, and the chi-square or Fisher’s exact test for categorical variables. All statistical analyses were performed using SAS^®^ version 9.4 (SAS Institute Inc., Cary, NC, USA).

## Results

### Participants

**Fig. 2:**
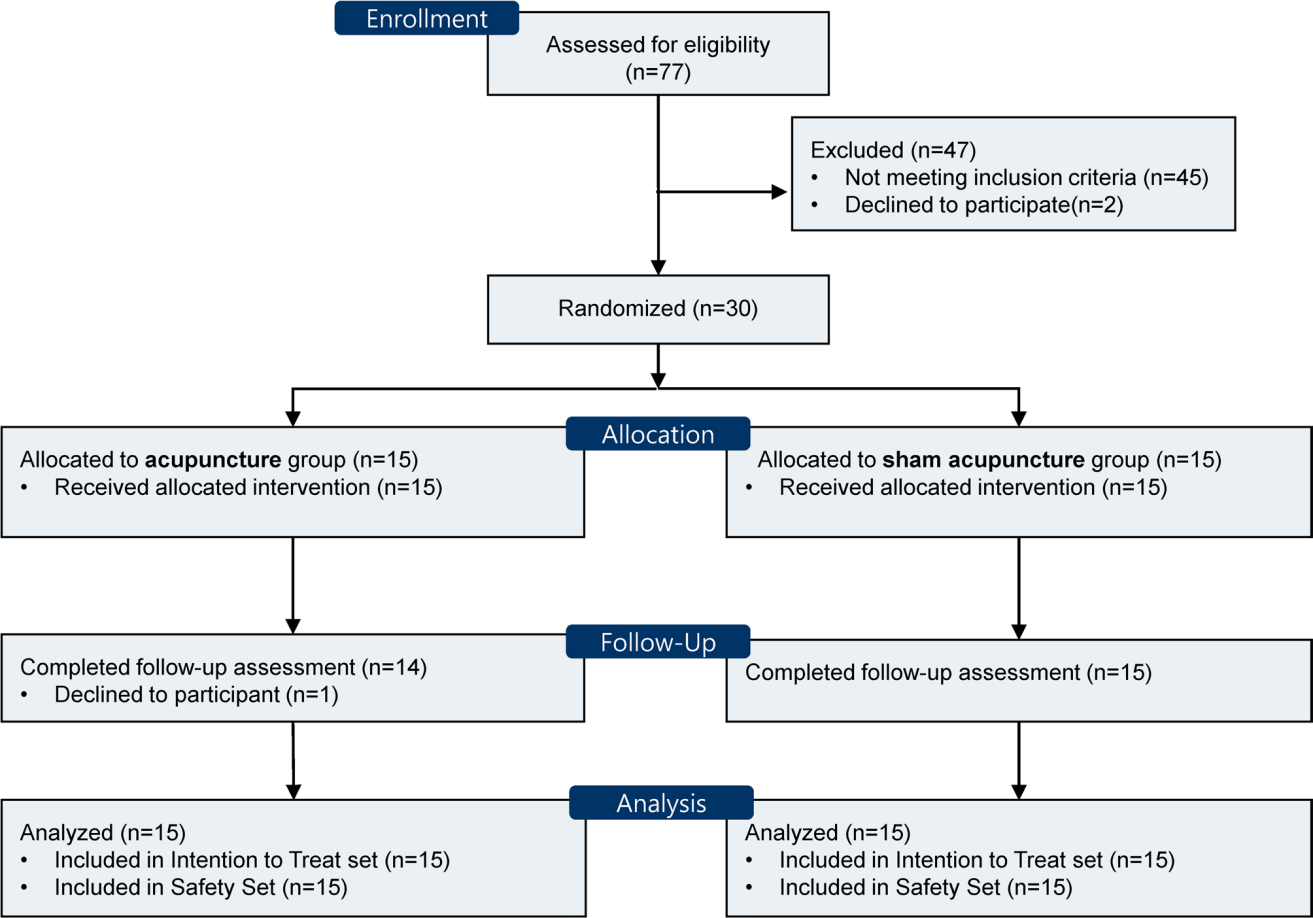
Flow diagram of the study

From December 2015 to September 2016, 77 participants were screened for eligibility, and 30 were enrolled in the trial. The enrolled participants were randomized into two groups: acupuncture (*n* = 15) and sham acupuncture (*n* = 15). One participant in the acupuncture group declined participation 6 weeks after baseline, and one participant in the sham acupuncture group did not attend the assessment at week 12. The remaining 28 participants completed the study (Fig. 2).

The demographic and baseline clinical characteristics of the enrolled participants are presented in Table 1. The mean age was 64.9 ± 7.2 and 64.6 ± 8.4 years in the acupuncture and sham acupuncture groups, respectively. Women accounted for 60.0% and 67.7% in the acupuncture and sham acupuncture groups, respectively. Education, employment, and medical histories were comparable between the two groups.

In both groups, the onset of cognitive problems occurred in the early 60s, and the participants had been experiencing cognitive issues for approximately 2–3 years. Baseline SNSB-II testing identified all enrolled participants with amnestic MCI and T-scores of ≤ 40 for the memory domain. In the acupuncture group, nine participants exhibited impairments only for the memory domain, with five participants exhibiting similar impairments in the sham acupuncture group. The remaining participants exhibited impairments in additional domains, such as frontal/executive function.

All participants exhibited Hachinski ischemic score of 0–3, excluding the possibility of vascular dementia, Clinical Dementia Rating scores of 0.5, and GDS scores of 2–3, indicating MCI. In the acupuncture group, 66.7% of the participants had GDS scores of 2 (very mild cognitive decline) compared with 46.7% in the sham acupuncture group, although the difference was not statistically significant. Additionally, participants in the acupuncture group exhibited a trend towards a lesser decline in MoCA and ADAS-cog scores, although this was not statistically significant.

**Table 1:** Patients’ demographic and clinical characteristics at baseline

		Acupuncture (*n* = 15)	Sham acupuncture (*n* = 15)	*P*-value
Age, yr	Mean ± SD	64.9 ± 7.2	64.6 ± 8.4	0.927
Sex, n (%)	Male	6 (40.0%)	5 (33.3%)	0.999
	Female	9 (60.0%)	10 (67.7%)	–
Education, yr	Mean ± SD	11.8 ± 4.5	10.6 ± 2.7	0.388
	≤ 6 years	3 (20.0%)	1 (6.7%)	0.165
	7–9 years	3 (20.0%)	7 (46.7%)	–
	10–12 years	3 (20.0%)	5 (33.3%)	–
	≥ 13 years	6 (40.0%)	2 (13.3%)	–
Employment status	Employed	5 (33.3%)	2 (13.3%)	0.388
	Unemployed	10 (66.7%)	13 (86.7%)	–
Age at onset of cognitive problems, yr	Mean ± SD	62.4 ± 7.2	61.7 ± 8.6	0.802
Duration of cognitive problems, yr	Mean ± SD	2.5 ± 1.1	2.8 ± 1.5	0.494
Medical history	Hypertension	6 (40.0%)	6 (40.0%)	0.999
	DM	0 (0.0%)	3 (20.0%)	0.224
	Hyperlipidemia	5 (33.3%)	3 (20.0%)	0.680
MCI subtypes	aMCI (single)	9 (60.0%)	5 (33.3%)	0.272
	aMCI (multiple)	6 (40.0%)	10 (66.7%)	–
Hachinski ischemic score	Mean ± SD	0.9 ± 0.7	1.3 ± 1.0	0.152
GDS	GDS 2	10 (66.7%)	7 (46.7%)	0.461
	GDS 3	5 (33.3%)	8 (53.3%)	–
ADAS-cog-11	Mean ± SD	10.3 ± 4.0	11.5 ± 3.9	0.411
MoCA	Mean ± SD	21.5 ± 3.8	19.5 ± 3.7	0.152

aMCI, amnestic MCI; DM, diabetes mellitus; GDS, Global Deterioration Scale; MCI, mild cognitive impairment; MoCA, Montreal Cognitive Assessment.

### Primary outcome

The ADAS-cog score decreased from 10.27 ± 4.03 and 11.47 ± 3.85 at baseline to 5.78 ± 3.04 and 6.27 ± 2.83 at week 12 in the acupuncture and sham acupuncture groups, respectively. After adjusting for baseline values, the mean difference between the two groups at 12 weeks was ˗0.49 (95% confidence interval [CI] ˗2.73, 1.76). Although the acupuncture group showed a slight tendency toward less cognitive decline, the difference was not statistically significant. Notably, Cohen’s d of 0.17 indicated a small effect size [36] (Table 2).

**Table 2:** Changes in clinical outcomes in the acupuncture and Sham acupuncture groups

	Acupuncture (*n* = 15)	Sham acupuncture (*n* = 15)	Mean difference (95% CI)	*P*-value	Cohen’s d	Effect size
**ADAS-cog-11 (range: 0–70)**						
Baseline	10.27 ± 4.03	11.47 ± 3.85				
Week 4	7.62 ± 2.70	8.32 ± 2.70	˗0.70 (˗2.75, 1.34)	0.4868	0.26	Small
Week 8	7.18 ± 2.94	7.01 ± 2.87	0.17 (˗2.04, 2.38)	0.8744	˗0.06	–
Week 12	5.78 ± 3.04	6.27 ± 2.83	˗0.49 (˗2.73, 1.76)	0.6590	0.17	Small
**MoCA (range: 0–30)**						
Baseline	21.47 ± 3.78	19.47 ± 3.66				
Week 4	24.16 ± 2.72	23.64 ± 2.72	0.52 (˗1.56, 2.61)	0.6098	0.19	Small
Week 8	24.90 ± 2.46	24.74 ± 2.44	0.15 (˗1.72, 2.03)	0.8664	0.06	–
Week 12	25.39 ± 2.89	25.79 ± 2.80	˗0.39 (˗2.57, 1.78)	0.7124	˗0.14	Small
**SNSB-II: Attention (T score)**						
Baseline	45.61 ± 9.84	46.91 ± 10.24				
Week 12	49.17 ± 10.99	49.50 ± 10.64	˗0.33 (˗8.46, 7.81)	0.9345	˗0.03	–
**SNSB II: Language and related functions (T score)**						
Baseline	45.81 ± 14.63	50.75 ± 9.28				
Week 12	52.74 ± 7.90	49.46 ± 7.69	3.29 (˗2.59, 9.17)	0.2605	0.42	Moderate
**SNSB-II: Visuospatial functions (T score)**						
Baseline	44.01 ± 14.07	46.52 ± 7.14				
Week 12	51.53 ± 7.22	50.06 ± 7.11	1.47 (˗3.92, 6.86)	0.5788	0.21	Small
**SNSB-II: Memory (T score)**						
Baseline	20.14 ± 8.73	20.09 ± 6.87				
Week 12	28.12 ± 5.05	25.30 ± 4.84	2.82 (˗0.91, 6.54)	0.1317	0.57	Moderate
**SNSB-II: Frontal/Executive functions (T score)**						
Baseline	44.51 ± 13.39	35.91 ± 12.70				
Week 12	48.93 ± 8.64	49.08 ± 8.07	˗0.16 (˗6.70, 6.39)	0.9611	˗0.02	–

ADAS-cog-11, 11 item Alzheimer’s Disease Assessment Scale-Cognitive Subscale; MoCA, Montreal Cognitive Assessment; SNSB-II, 2nd edition of the Seoul Neuropsychological Screening Battery.

### Secondary outcomes

No difference in MoCA scores between the acupuncture and sham acupuncture groups was observed at week 12. For the SNSB-II, the baseline scores for both groups were > 40 for the attention, language, and visuospatial function domains. For the memory domain, both groups showed clear impairments, with baseline T scores of approximately 20. After the 12-week intervention, the SNSB-II memory domain T score increased to 28.12 ± 5.05 and 25.30 ± 4.84 in the acupuncture and sham acupuncture groups, respectively, indicating a tendency for a greater increase in the acupuncture group. Although the P-value of 0.1317 indicated a non-statistically significant difference, Cohen’s d value of 0.57 suggested a moderate effect size [36] (Table 2).

After the 12-week intervention, the PGIC ratings in the acupuncture group were as follows: very much improved, *n* = 3; much improved, *n* = 3; minimally improved, *n* = 6; no change, *n* = 1; and minimally worse, *n* = 1. In the sham acupuncture group, the PGIC ratings were as follows: very much improved, *n* = 3; much improved, *n* = 2; minimally improved, *n* = 7; and no change, *n* = 2. The difference in the PGIC between the two groups was almost negligible (*P* = 0.9999), with approximately half of the participants in both groups reporting minimal improvement.

### Adverse events

In the acupuncture group, 7 of the 15 participants reported a total of 10 adverse events, none of which were related to the intervention. Acute nasopharyngitis was the most common adverse event, accounting for six cases. In the sham acupuncture group, 4 of 15 participants reported a total of eight adverse events, which were also not related to the intervention. Acute nasopharyngitis (*n* = 3) was the most common adverse event. No intervention-related adverse events were observed in either group (Table 3).

**Table 3:** Adverse events reported during the study

	Acupuncture (*n* = 15)	Sham acupuncture (*n* = 15)
**Severity of adverse events**		
Mild	6	7
Moderate	4	1
Severe	0	0
**Causality of adverse events**		
Definitely related	0	0
Probably related	0	0
Possibly related	0	0
Unlikely related	0	0
Definitely not related	10	8
Total number of participants with adverse events	7	4
Total number of adverse events	10	8
Total number of interventions	349	361

### Blinding test

A blinding test and credibility assessment of the treatment were performed to assess the success of blinding. The blinding index for the acupuncture group was 0.73 (95% CI 0.51, 0.94) at baseline and 1.00 (95% CI 1.00, 1.00) at week 12, indicating that participants in the acupuncture group believed they received classical acupuncture. For the sham acupuncture group, the blinding index was ˗0.27 (95% CI ˗0.70, 0.17) at baseline and ˗0.50 (95% CI ˗0.93, ˗0.07) at week 12, suggesting that participants in the sham acupuncture group also believed they received classical acupuncture (Table 4).

**Table 4:** Blinding test in the acupuncture and Sham acupuncture groups

		Acupuncture	Sham acupuncture	*P*-value
Baseline	Classical acupuncture	11 (73.33%)	8 (53.33%)	0.1693
	Non-classical acupuncture	0 (0.00%)	4 (26.67%)	–
	“Don’t know”	4 (26.67%)	3 (20.00%)	–
	Blinding index	0.73 (0.51, 0.94)	˗0.27 (˗0.70, 0.17)	–
Week 12	Classical acupuncture	14 (100.00%)	10 (71.43%)	0.0978
	Non-classical acupuncture	0 (0.00%)	3 (21.43%)	–
	“Don’t know”	0 (0.00%)	1 (7.14%)	–
	Blinding index	1.00 (1.00, 1.00)	˗0.50 (˗0.93, ˗0.07)	–

Regarding the credibility of the treatment results, participants in both groups responded with an average score of over 4 on the 7-point Likert scale (range: 0–6) for all four questions. No significant differences were observed between the groups, and the sham acupuncture group exhibited higher scores on some questions, indicating successful blinding in the sham acupuncture group (Table 5).

**Table 5:** Credibility of treatment rating scale in the acupuncture and Sham acupuncture groups

	Acupuncture	Sham acupuncture	Mean difference (95% CI)	*P*-value
**How confident do you feel that this treatment can alleviate your complaint? (range: 0–6)**				
Baseline	4.47 ± 1.41	4.13 ± 1.25	0.33 (˗0.66, 1.33)	0.4979
Week 12	4.29 ± 1.20	4.64 ± 1.34	˗0.36 (˗1.35, 0.63)	0.4642
**How confident would you be in recommending this treatment to a friend who suffered from similar complaints? (range: 0–6)**				
Baseline	4.53 ± 1.55	4.33 ± 1.18	0.20 (˗0.83, 1.23)	0.6938
Week 12	4.57 ± 0.94	5.07 ± 1.07	˗0.50 (˗1.28, 0.28)	0.2004
**How logical does this treatment seem to you? (range: 0–6)**				
Baseline	5.00 ± 1.25	4.27 ± 1.33	0.73 (˗0.24, 1.70)	0.1321
Week 12	5.07 ± 1.21	5.07 ± 1.00	0.00 (˗0.86, 0.86)	0.9999
**How successful do you think this treatment would be in alleviating other complaints? (range: 0–6)**				
Baseline	4.47 ± 1.41	4.47 ± 1.30	0.00 (˗1.01, 1.01)	0.9999
Week 12	4.50 ± 1.34	5.07 ± 0.83	˗0.57 (˗1.44, 0.30)	0.1875

## Discussion

### Summary of findings

This randomized pilot trial was designed to test the feasibility of a large-scale clinical trial. Notably, 30 participants were enrolled over a 10-month period, and 28 (93.3%) completed the 12-week treatment and evaluation period. All participants diagnosed with amnestic MCI, as indicated by SNSB-II memory domain T scores of ≤ 40, showed high compliance with the study protocol, with only one dropout in the acupuncture group and one missed evaluation in the sham acupuncture group. Further, a minimal mean difference in the ADAS-cog-11 score, the primary outcome, was observed between the two groups after 12 weeks of intervention. However, both groups showed clinically meaningful improvements of > 4 points compared with the baseline [37]. The SNSB-II memory domain T, the secondary outcome, was higher in the acupuncture group than in the sham acupuncture group, with a moderate effect size, despite not reaching statistical significance owing to the small sample size. No intervention-related adverse events were reported in either group. In this study, blinding was attempted using sham acupuncture in the control group, and the results of the blinding test and credibility assessment of the treatment revealed that participants in both groups believed that they received classical acupuncture treatment.

### Agreement and disagreement with previous literature

Most previous studies on acupuncture in patients with MCI have used the MoCA and MMSE as primary outcome measures [8, 9, 38]. However, we used MoCA as a secondary outcome, and the results revealed improvement in MoCA scores in the acupuncture and sham acupuncture groups after 12 weeks of treatment, with no significant difference between the groups. In contrast, a 2023 systematic review on acupuncture for patients with MCI, which synthesized results from seven studies using MoCA as an outcome measure, found acupuncture to be significantly more effective than control treatments [9]. Notably, all but one of these seven studies used medications such as nimodipine or donepezil as controls, with only one study employing sham acupuncture as a control. Additionally, three studies utilized manual acupuncture, similar to our study, and the others used electroacupuncture or warm acupuncture. Differences also existed in treatment frequency, with five studies administering treatments 5–6 times per week and the remaining two studies administering treatments every other day or three times per week [9]. Notably, the frequency of interventions, which was two times a week, was lower in our study. The absence of a significant difference in MoCA scores between the acupuncture and control groups in our study, unlike previous studies, may be attributed to several factors. Notably, the differences in the control groups used, the lower frequency of interventions, and the frequency of MoCA assessments (monthly in our study) may have led to learning effects, causing score improvements in both groups compared with those at the baseline [39].

Most previous acupuncture studies using the ADAS-cog have focused on patients with AD rather than those with MCI [40–43]. For instance, a clinical trial involving 87 patients with mild to moderate AD assigned to either an acupuncture treatment or a donepezil hydrochloride group showed significant improvement in ADAS-cog scores in the acupuncture group [40]. Similarly, a study comparing the effects of acupuncture and donepezil in patients with AD based on three clinical trials demonstrated more favorable results for the acupuncture group [41]. Conversely, two clinical trials using ADAS-cog for patients with MCI and employing electroacupuncture showed no significant difference compared with the control group [44, 45]. Therefore, ADAS-cog-11 may not be an effective tool for assessing the efficacy of acupuncture in patients with MCI. Our clinical trial used the ADAS-cog-11, which does not include tasks for evaluating delayed recall related to memory. For MCI clinical trials, the ADAS-cog-13, which includes word list delayed recall and number cancellation, is often used [46] and is better to measure MCI [47]. Despite the limitations, the acupuncture and sham acupuncture groups in our study showed improvements of > 4 points in ADAS-cog-11 scores compared with baseline. Notably, a change of 3 or 4 points on the ADAS-cog-11 is considered clinically meaningful [37, 48]. Therefore, the improvements observed in our study may be clinically relevant and warrant further investigation into the potential benefits of acupuncture in patients with MCI.

In our study, the effects of acupuncture compared with those of sham acupuncture were most notable for the SNSB-II memory domain T score, which showed a moderate effect size despite not reaching statistical significance, owing to the small sample size. This aligns with previous research indicating that acupuncture can affect memory-related brain regions. For instance, an fMRI connectivity analysis study found significant changes in functional connectivity in brain regions associated with memory encoding and retrieval (hippocampus, amygdala) following acupuncture compared with sham acupuncture [49]. Another study on patients with MCI and AD observed functional brain changes after acupuncture at LR3 and LI4, reporting activation in memory and cognition-related temporal and frontal lobes [50]. Additionally, in animal models of cognitive impairment, acupuncture inhibited cognition-related neuronal apoptotic pathways and contributed to the functional recovery of neurons in the hippocampal region, enhancing spatial learning and memory function [51].

The lack of significant differences between the acupuncture and sham acupuncture groups in the present study may be attributed to the inherent limitations of sham acupuncture. Although the non-penetrating Park sham device and non-acupoint locations achieved blinding, the stimulation provided by the sham device may have produced enhanced placebo and physiological effects partially resembling those of real acupuncture [52]. This could have reduced the differences between the groups, thereby making it more difficult to detect significant differences. Notably, previous studies have reported similar limitations, with a sham acupuncture device being more effective than an inert placebo pill [53] and even skin touch creating specific effects [54]. Our findings emphasize the methodological challenges in designing acupuncture trials and the need for careful interpretation of results in sham-controlled studies.

### Study limitations

Our study had some limitations. First, as this was a pilot feasibility trial, the results are preliminary and not conclusive. Further research with larger sample sizes and longer treatment durations is essential to comprehensively evaluate the efficacy and safety of acupuncture for MCI. Additionally, confirmatory trials are necessary to validate our hypothesis that 12 weeks of acupuncture treatment can improve memory compared with sham acupuncture.

Second, the study did not include a long-term follow-up period, which is essential for assessing the long-term effects of acupuncture on preventing progression to dementia [55]. Owing to time and budget constraints, the study was concluded after the 12-week treatment and assessment period, and the long-term effects of acupuncture were not evaluated.

Third, the study used the ADAS-cog-11, which may not be the most effective tool for assessing the efficacy of acupuncture in patients with MCI, as it does not include tasks for evaluating delayed recall related to memory. The ADAS-cog-13, which includes additional tasks such as word list delayed recall and number cancellation [27], is often more appropriate for MCI clinical trials and better assesses MCI [56]. Additionally, the monthly assessments using MoCA could have led to learning effects [39], causing score improvements in both groups when compared with those at baseline and masking any potential differences between the acupuncture and sham acupuncture groups.

Finally, the frequency of acupuncture treatment in this study was two times weekly, which is lower than the frequency used in other studies (5–6 times weekly) [8, 9, 38], which may have contributed to the lack of significant differences between the acupuncture and sham acupuncture groups.

### Implication for further studies

The sample size for a confirmatory trial could be calculated based on the estimated effect size of acupuncture compared with sham acupuncture in this pilot trial. Using Cohen’s d of 0.57 for the SNSB-II memory T score, the required sample size is 39 for each group [57]. Raw scores from the SVLT-E [32] and RCFT [31] used to calculate the SNSB-II memory domain T score could also be utilized (Table 6). Based on the results of this pilot trial, the SNSB-II memory T score or SVLT-E delayed recall score could be considered the primary outcomes. Depending on the research objectives, the inclusion criteria could be limited to patients with amnestic MCI to recruit a more homogeneous participant group.

**Table 6:** Calculated sample size for further research based on the estimated effect size in SNSB-II memory, SVLT-E, and RCFT results

	Acupuncture (*n* = 15)	Sham acupuncture (*n* = 15)	Mean difference (95% CI)	*P*-value	Cohen’s d	*n* (each group)
**SNSB-II: Memory (T score)**						
Baseline	20.14 ± 8.73	20.09 ± 6.87	–	–	–	–
Week 12	28.12 ± 5.05	25.30 ± 4.84	2.82 (˗0.91, 6.54)	0.1317	0.57	38.75
**SVLT-E immediate recall total (range: 0–36)**						
Baseline	16.13 ± 3.52	15.20 ± 3.95	–	–	–	–
Week 12	18.91 ± 5.28	17.36 ± 5.28	1.55 (˗2.44, 5.54)	0.4314	0.29	147.71
**SVLT-E delayed recall (range: 0–12)**						
Baseline	4.80 ± 2.14	3.67 ± 1.72	–	–	–	–
Week 12	4.84 ± 2.45	3.49 ± 2.30	1.35 (˗0.50, 3.20)	0.1436	0.57	38.75
**RCFT immediate recall (range: 0–36)**						
Baseline	15.67 ± 6.70	18.07 ± 5.71	–	–	–	–
Week 12	20.79 ± 4.85	19.28 ± 4.73	1.51 (˗2.12, 5.14)	0.3997	0.32	121.44
**RCFT delayed recall (range: 0–36)**						
Baseline	13.73 ± 5.18	16.67 ± 5.90	–	–	–	–
Week 12	20.55 ± 4.00	18.92 ± 3.90	1.62 (˗1.40, 4.64)	0.2783	0.41	74.24

SNSB-II, 2nd edition of the Seoul Neuropsychological Screening Battery; SVLT-E, Seoul Verbal Learning Test, elderly version; RCFT, Rey Complex Figure Test.

The high retention rate (93.3%) and treatment adherence (97–100%) were observed in this pilot study. While our pilot study achieved these favorable outcomes, confirmatory trials typically experience higher dropout rates due to multi-center implementation, larger sample sizes, and more diverse patient populations. Based on systematic analysis of MCI trials showing dropout rates of 8.1% for 6-month and 15.8% for 12-month studies [58], we recommend planning for a conservative 10–15% dropout rate in future confirmatory trials and can expect similar high compliance rates with twice-weekly treatment schedules. Based on these considerations, a confirmatory trial would require approximately 44–46 participants per group (accounting for 10–15% dropout) and could achieve target enrollment within 12–18 months using a multi-center approach.

Currently, consensus on common evaluation metrics for non-pharmacological intervention trials in MCI remains lacking [59]. The ADAS-cog remains a valuable candidate for primary outcome consideration considering its frequent use in previous MCI clinical trials [46, 59]. Although the ADAS-cog-13 could also be considered, the lack of an estimated effect size for acupuncture necessitates an additional pilot trial for an accurate sample size calculation. Additionally, further studies should consider incorporating objective biomarkers [60] and neuroimaging techniques [61] to explore potential neurobiological pathways through which acupuncture influences cognitive function in patients with MCI.

Increasing the frequency of interventions may be beneficial in future studies. In the current setting of twice-weekly treatments, all but 2 of the 30 participants completed the planned 24 sessions. Notably, increasing the frequency may enhance the efficacy of the intervention [62, 63]. Additionally, previous randomized controlled trials on the effects of acupuncture for MCI have conducted treatments 5–6 times per week [8, 9, 38]. However, the increased time and cost associated with more frequent sessions should be considered and adjusted for feasibility, particularly in the context of the healthcare system and clinical setting in South Korea. Higher treatment frequencies may potentially enhance the effects of acupuncture; however, the practicality of having patients attend sessions five times weekly for 3 months should be carefully considered.

The sham acupuncture method used in this study involved non-penetrating the skin with the Park sham device [24] and non-acupoint locations for stimulation. The adequate blinding achieved in this study, as evidenced by two separate tests, suggests that similar methods can be effectively used in future trials to establish a control group. However, the stimulation provided by the Park sham device, although helpful for blinding, may have produced physiological effects that partially resembled the effects of real acupuncture [52], reducing the observed differences between the two groups. Future studies should consider these limitations while designing sham-controlled acupuncture trials.

Future confirmatory trials should include sufficient follow-up periods to assess the long-term effects of acupuncture on MCI. A sham-controlled acupuncture study on knee osteoarthritis that found no significant differences between groups during the treatment period revealed that the effects of real acupuncture were better maintained during the follow-up period, whereas the effects of sham acupuncture tended to return to baseline levels [64]. Owing to the pilot nature of our study and design constraints, we did not include a follow-up period. Future studies should incorporate follow-up assessments to evaluate the potential long-term benefits of acupuncture in patients with MCI.

## Conclusion

This pilot, randomized, controlled trial showed promising results regarding the feasibility of conducting a large-scale clinical trial to evaluate the efficacy and safety of acupuncture for MCI. This study observed clinically meaningful improvements in the acupuncture and sham acupuncture groups, with a moderate effect size favoring acupuncture based on the SNSB-II memory domain T score. Adequate blinding was achieved using a non-penetrating sham acupuncture device and non-acupoint locations. However, the absence of significant differences between the groups may be attributed to the limitations of sham acupuncture, lower frequency of interventions, and small sample size. Based on the preliminary findings of this pilot trial, further confirmatory trials should be planned to test the effects of acupuncture on memory in patients with MCI.

## Electronic supplementary material

Below is the link to the electronic supplementary material.

Supplementary Material 1

Supplementary Material 2

Supplementary Material 3

Supplementary Material 4

## Data Availability

The datasets used and analysed during the current study are available from the corresponding author on reasonable request.

## Abbreviations

MCI: Mild cognitive impairment
AD: Alzheimer’s disease
GDS: Global Deterioration Scale
ADAS-cog: Alzheimer’s Disease Assessment Scale-Cognitive Subscale
MoCA: Montreal Cognitive Assessment
SNSB-II: 2nd edition of the Seoul Neuropsychological Screening Battery
PGIC: Patient Global Impression of Change
RCFT: Rey Complex Figure Test
SVLT-E: Seoul Verbal Learning Test, elderly version
