# Supplementary material for: Acupuncture for patients with mild cognitive impairment: a randomized, patient–assessor-blinded, sham-controlled pilot study
Source: BMC Complement Med Ther. 2025 Jul 19;25:277. doi: 10.1186/s12906-025-05023-5 (PMC12275449; doi:10.1186/s12906-025-05023-5)
Supplement: Supplementary file 2 — Supplementary Material 2 [file 12906_2025_5023_MOESM2_ESM.docx]

**Supplementary File 2.** The STRICTA checklist

| **Item** | **Description** | **Details in this study** |
| --- | --- | --- |
| **1. Acupuncture rationale** | 1a) Style of acupuncture (e.g. Traditional Chinese Medicine, Japanese, Korean, Western medical, Five Element, ear acupuncture, etc) | Based on Traditional Korean medicine |
|  | 1b) Reasoning for treatment provided, based on historical context, literature sources, and/or consensus methods, with references where appropriate | Based on previous research [1–3], 14 acupuncture points were selected by consensus from 5 Korean medicine doctor specialists. |
|  | 1c) Extent to which treatment was varied | Standardized acupuncture treatment was administered. |
| **2. Details of needling** | 2a) Number of needle insertions per subject per session (mean and range where relevant) | 14 acupuncture needles |
|  | 2b) Names (or location if no standard name) of points used (uni/bilateral) | GV20, EX-HN1, CV12, and bilateral points of ST36, HT7, KI3, and SP6 |
|  | 2c) Depth of insertion, based on a specified unit of measurement, or on a particular tissue level | 0.2–1.5 cun |
|  | 2d) Response sought (e.g. *de qi* or muscle twitch response) | Localized de qi sensation |
|  | 2e) Needle stimulation (e.g. manual, electrical) | Manual acupuncture |
|  | 2f) Needle retention time | 30 minutes |
|  | 2g) Needle type (diameter, length, and manufacturer or material) | Stainless steel, size 0.25 x 40 mm, Dongbang Medical Co., Ltd., Republic of Korea |
| **3. Treatment regimen** | 3a) Number of treatment sessions | 24 sessions |
|  | 3b) Frequency and duration of treatment sessions | 12 weeks (twice a week) |
| **4. Other components of treatment** | 4a) Details of other interventions administered to the acupuncture group (e.g. moxibustion, cupping, herbs, exercises, lifestyle advice) | Other interventions were restricted during the study period. |
|  | 4b) Setting and context of treatment, including instructions to practitioners, and information and explanations to patients | Practitioners were allowed to converse with participants only as necessary for treatment, while all unrelated conversation was prohibited. |
| **5. Practitioner background** | 5) Description of participating acupuncturists (qualification or professional affiliation, years in acupuncture practice, other relevant experience) | Qualified Korean Medicine Doctor with at least 2 years of clinical experience |
| **6. Control or comparator interventions** | 6a) Rationale for the control or comparator in the context of the research question, with sources that justify this choice | Sham acupuncture was used to exclude placebo effect [4]. |
|  | 6b) Precise description of the control or comparator. If sham acupuncture or any other type of acupuncture-like control is used, provide details as for Items 1 to 3 above. | Sham acupuncture was administered using a non-penetrating Park sham device [5] at non-acupoints, with the same frequency, duration, and method as the acupuncture group. |

**References**

1. Zhou J, Peng W, Xu M, Li W, Liu Z. The effectiveness and safety of acupuncture for patients with Alzheimer disease: a systematic review and meta-analysis of randomized controlled trials. Medicine. 2015;94(22):e933.

2. Jia B, Liu Z, Min B, Wang Z, Zhou A, Li Y, et al. The effects of acupuncture at real or sham acupoints on the intrinsic brain activity in mild cognitive impairment patients. Evidence-Based Complementary and Alternative Medicine. 2015;2015.

3. Leung AWN, Lam LCW, Kwan AKL, Tsang CLL, Zhang HW, Guo YQ, et al. Electroacupuncture for older adults with mild cognitive impairment: study protocol for a randomized controlled trial. Trials [Internet]. 2015 May 27 [cited 2019 Sep 18];16. Available from: https://www.ncbi.nlm.nih.gov/pmc/articles/PMC4451728/

4. Vincent CA. The Methodology of Controlled Trials of Acupuncture. Acupunct Med. 1989 Jan 1;6(1):9–13.

5. Park J, White A, Stevinson C, Ernst E, James M. Validating a New Non-Penetrating Sham Acupuncture Device: Two Randomised Controlled Trials. Acupunct Med. 2002 Dec;20(4):168–74.
