# Supplementary material for: Acupuncture for patients with mild cognitive impairment: a randomized, patient–assessor-blinded, sham-controlled pilot study
Source: BMC Complement Med Ther. 2025 Jul 19;25:277. doi: 10.1186/s12906-025-05023-5 (PMC12275449; doi:10.1186/s12906-025-05023-5)
Supplement: Supplementary file 3 — Supplementary Material 3 [file 12906_2025_5023_MOESM3_ESM.docx]

Supplementary File 3. The ACURATE checklist

| **Category** | **Item** | **Description** | **Details in this study** |
| --- | --- | --- | --- |
| 1. Type of sham acupuncture | 1a | Report the type of sham acupuncture. | Non-penetrating sham acupuncture using Park sham device [1]. |
|  | 1b | Report whether the sham acupuncture is penetrating or non-penetrating. | Non-penetrating |
|  | 1c | Rationale for using the chosen sham acupuncture. | To minimize the active effect of sham acupuncture, a non-penetrating type was chosen. |
| 2. Details of sham acupuncture manipulation | 2a | Report the number of sham acupuncture applied per subject per session. | 14 sham acupuncture devices |
|  | 2b | Report the depth of sham acupuncture insertion (if there was no penetration, state this within the paper). | No penetration |
|  | 2c | Report whether any response was observed during sham acupuncture manipulation (e.g. de qi or muscle twitch response). | De qi sensation was not obtained |
|  | 2d | Report if there was any stimulation using sham acupuncture. | No stimulation was obtained |
|  | 2e | Report if there was sham acupuncture retention. | 30 minutes |
|  | 2f | Report details of other interventions administered in addition to sham acupuncture during one session. | Other interventions were not administered |
| 3. Location of sham acupuncture | 3a | Report the location of sham acupuncture (e.g. acupoint/non-acupoint or the exact location of the sites). | 14 non-acupoints: the midpoint of the biceps brachii muscle belly (Upper Extremity 1, UE1); 2 cm above UE1 (UE2); 5 cm below the elbow crease and 1 cm lateral (UE3); 2 cm above UE3 (UE4); the upper third of the medial part of the tibia (Lower Extremity 1, LE1); 2 cm below LE1 (LE2); and 2 cm below LE2 (LE3), all bilaterally. |
|  | 3b | Explicitly state in the paper if the points are unilateral or bilateral. | Bilateral |
|  | 3c | Describe the reason for the chosen location of sham acupuncture. | 1) locations that do not correspond to recognized traditional acupoints; 2) areas without established therapeutic effects for cognitive function in the literature; and 3) considering that dermatome overlap between verum and sham acupuncture can influence clinical outcomes, dermatome overlap was minimized where anatomically feasible. |
| 4. Treatment regimen | 4a | Report the number of treatment sessions. | 24 sessions |
|  | 4b | Report whether the number of sessions were identical between real and sham acupuncture treatments. | Identical |
|  | 4c | Report the frequency and duration of treatment sessions. | 12 weeks (twice a week) |
|  | 4d | Report the total trial period. | About 13 weeks, including screening period. |
| 5. Practitioner | 5a | Report whether the same practitioner is administering both real and control treatments (interventions). | Same practitioners administered both real and sham acupuncture. |
|  | 5b | Report whether there were conversations between practitioner and patient directly linked to the trial design, other than scripted instructions and preset information, prior to and during the treatment. | Practitioners were allowed to converse with participants only as necessary for treatment, while all unrelated conversation was prohibited. |
| 6. Protocol and settings | 6a | Report the information regarding sham acupuncture provided to participants. | For patient blinding, participants were informed that they would receive one of two types of acupuncture treatments: "classical acupuncture" or "non-classical acupuncture." |
|  |  | Report whether the information given to patients include the term to openly state that the control is inert (e.g. "fake", "sham", "dummy","placebo", ...). |  |
|  | 6b |  |  |
|  | 6c | Describe how sham device was blinded from patients, and if done, how the blinding was assessed. | To assess the success of blinding, participants were asked to guess which treatment they received after the first and last interventions [2]. They could choose from: “classical acupuncture,” “non-classical acupuncture,” or “don't know which treatment I received.” Additionally, the credibility of treatment was evaluated at the end of the 12-week treatment period [3]. Participants rated their responses to four questions using a 7-point Likert scale (0 = very low to 6 = very high): “How confident do you feel that this treatment can alleviate your complaint?”; “How confident would you be in recommending this treatment to a friend who suffered from similar complaints?”; “How logical does this treatment seem to you?”; and “How successful do you think this treatment would be in alleviating other complaints?” |
|  | 6d | If done, report any modification in the sham acupuncture treatment procedure, and reason for the modification. | Not applicable |
|  | 6e | Report any difference in the treatment settings between real and sham acupuncture. | There was no difference. |

**References**

1. Park J, White A, Stevinson C, Ernst E, James M. Validating a New Non-Penetrating Sham Acupuncture Device: Two Randomised Controlled Trials. Acupunct Med. 2002 Dec;20(4):168–74.

2. Bang H, Ni L, Davis CE. Assessment of blinding in clinical trials. Controlled clinical trials. 2004;25(2):143–56.

3. Vincent C, Lewith G. Placebo controls for acupuncture studies. J R Soc Med. 1995 Apr;88(4):199–202.
