# Supplementary material for: Acupuncture for patients with mild cognitive impairment: a randomized, patient–assessor-blinded, sham-controlled pilot study
Source: BMC Complement Med Ther. 2025 Jul 19;25:277. doi: 10.1186/s12906-025-05023-5 (PMC12275449; doi:10.1186/s12906-025-05023-5)
Supplement: Supplementary file 4 — Supplementary Material 4 [file 12906_2025_5023_MOESM4_ESM.docx]

# Table S1. Results of repeated measure ANOVA in ADAS-cog 11 and MoCA

|  |  | **df** | ***F*** | **p-value** |
| --- | --- | --- | --- | --- |
| ADAS-cog-11 (range: 0–70) | group | (1, 27) | 0.12 | 0.732 |
|  | time | (3, 81) | 21.78 | <0.0001 |
|  | group:time | (3, 81) | 0.33 | 0.804 |
| MoCA (range: 0–30) | group | (1, 27) | 0.25 | 0.621 |
|  | time | (3, 81) | 43.07 | <0.0001 |
|  | group:time | (3, 81) | 0.72 | 0.542 |

ANOVA, analysis of variance; ADAS-cog-11, 11 item Alzheimer's Disease Assessment Scale-Cognitive Subscale; MoCA, Montreal Cognitive Assessment.

**Table S2. List of reported adverse events**

| **Subject ID** | **Group** | **Adverse event** | **Duration (day)** | **Severity** | **Results** | **Causality** | **Action taken** |
| --- | --- | --- | --- | --- | --- | --- | --- |
| 1001 | Acupuncture | Hypertension | - | moderate | not recovered or not resolved | Definitely not related | concomitant medication administration |
|  |  | Common cold | 1 | mild | recovered or resolved | Definitely not related | concomitant medication administration |
|  |  | Rib pain | 15 | moderate | recovered or resolved | Definitely not related | concomitant medication administration |
| 1004 | Acupuncture | Eyelid injury | 22 | moderate | recovered or resolved | Definitely not related | concomitant medication administration |
|  |  | Common cold | 7 | mild | recovered or resolved | Definitely not related | concomitant medication administration |
| 1005 | Acupuncture | Common cold | 8 | moderate | recovered or resolved | Definitely not related | concomitant medication administration |
| 1007 | Acupuncture | Common cold | 6 | mild | recovered or resolved | Definitely not related | concomitant medication administration |
| 2003 | Acupuncture | Common cold | 7 | mild | recovered or resolved | Definitely not related | concomitant medication administration |
|  |  | Chest discomfort | 0 | mild | recovered or resolved | Definitely not related | none |
| 2010 | Acupuncture | Common cold | 2 | mild | recovered or resolved | Definitely not related | concomitant medication administration |
| 2015 | Acupuncture | Eczema (face) | 13 | mild | recovered or resolved | Definitely not related | concomitant medication administration |
| 1003 | Sham acupuncture | Common cold | 29 | moderate | recovering or resolving | Definitely not related | concomitant medication administration |
|  |  | Common cold | 6 | mild | recovered or resolved | Definitely not related | concomitant medication administration |
| 1008 | Sham acupuncture | Common cold | 6 | mild | recovered or resolved | Definitely not related | concomitant medication administration |
|  |  | Periodontal disease | 6 | mild | recovered or resolved | Definitely not related | concomitant medication administration |
|  |  | Shoulder pain | 36 | mild | recovering or resolving | Definitely not related | concomitant medication administration |
| 2002 | Sham acupuncture | Food allergy | 1 | mild | recovered or resolved | Definitely not related | concomitant medication administration |
| 2004 | Sham acupuncture | Periodontal disease | 32 | mild | recovered or resolved | Definitely not related | concomitant medication administration |
|  |  | Toothache | 6 | mild | recovered or resolved | Definitely not related | concomitant medication administration |
